# The media and access issues: content analysis of Canadian newspaper coverage of health policy decisions

**DOI:** 10.1186/s13023-015-0320-z

**Published:** 2015-08-25

**Authors:** Christen Rachul, Timothy Caulfield

**Affiliations:** School of Linguistics and Language Studies, Carleton University, Paterson Hall 236, 1125 Colonel By Drive, Ottawa, ON K1S 5B6 Canada; Faculty of Law and School of Public Health, Health Law Institute, University of Alberta, Office 468, Edmonton, AB T6G 2H5 Canada

**Keywords:** Access to healthcare, News media, Canada

## Abstract

**Background:**

Previous studies have demonstrated how the media has an influence on policy decisions and healthcare coverage. Studies of Canadian media have shown that news coverage often emphasizes and hypes certain aspects of high profile health debates. We hypothesized that in Canadian media coverage of access to healthcare issues about therapies and technologies including for rare diseases, the media would be largely sympathetic towards patients, thus adding to public debate that largely favors increased access to healthcare—even in the face of equivocal evidence regarding efficacy.

**Methods:**

In order to test this hypothesis, we conducted a content analysis of 530 news articles about access to health therapies and technologies from 15 major Canadian newspapers over a 10-year period. Articles were analyzed for the perspectives presented in the articles and the types of reasons or arguments presented either for or against the particular access issue portrayed in the news articles.

**Results:**

We found that news media coverage was largely sympathetic towards increasing healthcare funding and ease of access to healthcare (77.4 %). Rare diseases and orphan drugs were the most common issues raised (22.6 %). Patients perspectives were often highlighted in articles (42.3 %). 96.8 % of articles discussed why access to healthcare needs to increase, and discussion that questioned increased access was only included in 33.6 % articles.

**Conclusion:**

We found that news media favors a patient access ethos, which may contribute to a difficult policy-making environment.

## Background

*“…unless those of us who believe in Medicare raise our voices in no uncertain terms, unless we arouse our neighbours and our friends and our communities, we are sounding the death knell of Medicare in this country.”*—Tommy Douglas

In 1984, Tommy Douglas, known as the father of Canadian Medicare, made a speech intended to encourage the Canadian population to defend the universal healthcare coverage for which he so vigorously fought in the 1960s [1]. Since this speech, Canadians have continued to raise their voices to express their views about what should be covered by our system [2].

The media have played an important part in this public dialogue. Media representations can play a role in the healthcare system including, for example, increasing public interest in and utilization of a particular service [3, 4] and complicating (and, perhaps, clouding) debates about public health policy issues [5]. There are also examples of situations where media coverage seems to have had a tangible impact on how the policy debates associated with funding decisions are framed, play out, and are ultimately resolved [6–8]. One study has articulated concern that media tends to favor a “rule of rescue” ethos [8]—that is, a perceived need to provide treatment to an individual with little concern for cost or other concerns [9].

In the context of access to healthcare, the Canadian media have tended to cover systemic issues such as wait times, doctor shortages and user fees [2]. However, more recently there have been several high profile cases in Canada that demonstrate increasing media coverage of access issues that relate to availability and coverage of health therapies and technologies. The framing of these stories in media coverage help emphasize certain aspects of a debate over others [10]. For example, Quebec’s 2009 decision to fund IVF treatments and more recent debates about coverage for costly cancer medications demonstrate the increasingly complex, and economically challenging, issues that face Canadians, and also highlights regional inequality in what has been called the “postal code lottery” [11, 12]. One research study also found that Canadian media coverage of the cancer drug Herceptin was more “hyped” in comparison to media portrayals from the UK, and that these studies focused on how long it took for drugs to receive funding approval, while downplaying taxpayer costs in favor of patients’ needs [13].

In addition to the role of media representations in policy decisions, it has been shown that the media can both reflect and shape public opinion [2, 5, 14]. Health news has also been shown to affect public perceptions of the seriousness of diseases and health issues [15] and even affect health-related behaviors [16, 17]. The popular media is one of the primary sources for health and science information [18] and healthcare dominates the health-related news stories in Canada [19]. As such, an examination of the news media portrayals of access issues that include availability and coverage of therapies and technologies and the “right” to healthcare in Canada—a concept that remains legally contentious [20, 21] – may provide both insight relevant to the policy-making process and a unique perspective on the framing of public opinion. Previous studies have demonstrated that popular media can be imbalanced and omit important information about risks and limitations, among other types of information [4, 13, 22–24]. How, then, does the media approach issues of access to therapies and technologies?

We conducted a descriptive content analysis of print news articles from major newspapers across Canada. We hypothesized that the media would be largely sympathetic towards patients, thus adding to public debate that largely favors increased access to an increasing list of therapies and technologies. Even in the face of equivocal evidence regarding efficacy, media portrayals that support increased access to healthcare confirms a patient access ethos in the Canadian media. In order to test this hypothesis, we examined articles for the perspectives presented in the articles and the types of reasons or arguments presented either for or against the particular access issue portrayed in the news articles.

## Methods

The descriptive content analysis was based on the notion that the framing of news media, or the central organizing ideas in a news article, can highlight certain aspect of a debate over others [10]. In turn, news media framing plays a role in how the public views an issue and how policy-makers define and understand their policy options [25]. In order to examine how Canadian media portrays issues of access or the right to healthcare in terms of therapies and technologies, an inductive approach to a content analysis was taken which began with a pre-determined list of variables and categories for each variable were developed through a process of constant comparison between news articles.

The data set included English language print news articles available on the Factiva and Canadian Newsstand databases. News articles were gathered from January 1, 2003 to December 31, 2012, which were published in the newspaper with top circulation in major cities from across Canada, including two national papers (Table 1) [26]. The data set was collected in two stages: 1) A search of relevant Canadian case law (e.g., see cases highlighted in the introduction) and a literature review were conducted to determine search terms that were relevant to news stories about access to healthcare in a Canadian context. These terms include variations of rare disease, orphan drugs, prostrate-specific antigen (PSA) testing, autism, and fertility and in vitro fertilization (IVF); 2) To include other issues not represented by court cases from 2003 to 2012, a search for articles with terms such as treatment cost, medically necessary, and drug funding were also used. While these search terms did not yield a comprehensive set of articles that address every issue of access to healthcare, they identified articles that are representative of the key social issues associated with patient access to therapies and technologies in Canada. Because we used well-known case law as a foundation for our search terms, we were able to identify media representations that were most relevant to funding decisions associated with the tension between patient needs and government policy. Irrelevant articles were then excluded from the data set, resulting in a final data set of 530 print news articles from both searches. Articles were deemed irrelevant or excluded if they were letters to the editor, classifieds, articles that mentions healthcare challenges that were not framed as access, funding or rights issues. Because we were interested in access to particular technologies or therapies, the sample also excludes systemic access issues such as doctor shortages and wait lists.

**Table 1:** Newspapers included in the sample

Newspaper	# of articles	% of data set
The Globe and Mail	86	16.2 %
The National Post	31	5.8 %
The Vancouver Sun	43	8.1 %
Victoria Times Colonist	10	1.9 %
Calgary Herald	48	9.1 %
Edmonton Journal	29	5.5 %
Saskatoon Star Phoenix	15	2.8 %
Regina Leader Post	5	0.9 %
Winnipeg Free Press	17	3.2 %
The Hamilton Spectator	34	6.4 %
Ottawa Citizen	70	13.2 %
The Toronto Star	85	16.0 %
Montreal Gazette	41	7.7 %
Daily Gleaner (Fredericton)	9	1.7 %
Telegraph-Journal (St. John)	7	1.3 %

The resulting data set of news articles were then coded by a single coder using a descriptive coding framework that was developed using methods from previous studies conducted by our team [24, 27]. The coding framework included both deductive and inductive items. The inductive items elicited descriptive codes for key variables, which included identifying which issue or issues were discussed in the article, from whose perspective the issue was discussed, as well as describing the reasons presented in favor of or opposition to greater access, and the evidence cited to support positions. The responses for inductive items were developed through a process of constant comparison throughout the coding process and then quantified for further analysis. Deductive items included descriptive information about the news article (e.g., newspaper, date published) and the overall tone of the article. To determine reliability of inductive and deductive coding, a second coder coded a random sample of 20 % of news articles and inter-coder agreement was calculated using Cohen’s Kappa (*k*). Inter-coder agreement ranged from *k* = 0.611 to 1.000, with an average of *k* = 0.744 indicating good to very good agreement.

## Results

The news articles covered a wide variety of issues, and included discussion of a range of conditions and issues relevant to specific populations. Articles were coded for up to two diseases, conditions, or access issues highlighted in the article. Rare diseases and orphan drugs comprised the most common issue that appeared in news articles from 2003 to 2012 (22.6 % of articles), but altogether a list of 26 different diseases, conditions or access issues were identified in 404 news articles (76.2 %) (Table 2). 126 of the articles (23.8 %) did not focus on a specific disease, condition or issue but did discuss issues of access to healthcare in more general terms. Almost half of the articles (252 articles, 47.5 %) focused on a particular population, for example minors (90 articles, 17.0 %), immigrants and refugees (25 articles, 4.7 %), or low-income or homeless populations (24 articles, 4.5 %).

**Table 2:** Top ten diseases, conditions, or issues identified in news articles

	# articles	% of articles
Rare disease/orphan drugs	120	22.6 %
Autism therapy	75	14.2 %
Cancer treatment	63	11.9 %
Fertility/IVF	36	6.8 %
Screening/testing	24	4.5 %
Infectious disease (e.g., HIV/AIDS, hepatitis, H1N1)	19	3.6 %
Chronic diseases (non-rare)	14	2.6 %
Mental health	11	2.1 %
Persons with disabilities (intellectual, physical, etc.)	11	2.1 %
Institutional/policy/physical barriers to access	8	1.5 %

The perspective from which the article was written provides insight into how the media frames controversies associated with access to healthcare in Canada. Nearly half of the articles were written from a patient's or caregiver’s perspective (224 articles, 42.3 %). Other perspectives were from healthcare providers (59 articles, 11.1 %), a provincial government (56 articles, 10.6 %), and the general public (45 articles, 8.5 %). While several other perspectives were provided—such as from scientists and other academic researchers—they appeared in only a handful of articles.

### Access to therapies and technologies in Canada

A primary issue covered by news articles focused on the appropriateness of current healthcare coverage policies for therapies and technologies (291 articles, 54.9 %). Other issues included the high cost of treatments (94 articles, 17.1 %), treatments being unavailable or having limited availability (56 articles, 10.6 %), and issues of policy development (52 articles, 9.8 %) (Table 3).

**Table 3:** Top ten access issues covered in news articles

	# articles	% of articles
Scope of government healthcare coverage	291	54.9 %
Barriers caused by high cost of treatment/service	94	17.7 %
Treatment/service is unavailable	56	10.6 %
Call for health policy reforms (e.g., orphan drug and catastrophic drug programs)	52	9.8 %
Legal proceedings (e.g., court decision relevant to access)	49	9.2 %
Access issues related to communication (e.g., access in language of choice)	27	5.1 %
Need for more research on rare diseases	24	4.5 %
Access issues related to transportation (e.g., no public transportation, service too far away)	22	4.2 %
Public health initiatives aimed at increased access (e.g., vaccine programs, nutrition education)	20	3.8 %
Health-related advocacy, fundraising, raising awareness about issues relevant to access	19	3.6 %

Not surprisingly, given the focus on government health coverage, the main theme of each article largely concerned attitudes towards the government. For example, themes included the government’s responsibility to improve access to healthcare (103 articles, 19.4 %), the government letting its citizens and residents down by not providing access (76 articles, 14.3 %), and inequality in the healthcare system (56 articles, 10.6 %) (Table 4).

**Table 4:** Top ten themes of news articles with example quotes

	# articles	% of articles
Access needs to be improved or maintained	103	19.4 %
“…she is fighting to maintain access to the treatment that allows her not only to live, but to take part in a regular exercise program and get some fun out of life.” [30]		
Government is letting citizens/residents down	76	14.3 %
“The Lindberg family is now attempting a round of aggressive antibiotics—all of it privately funded as the Alberta health system refuses to acknowledge the diagnosis of Lyme disease, she said.” [31]		
Patient needs are priority	71	13.4 %
“Pregnant women and their babies are among those experiencing the worst fallout from Ottawa's decision to scale back funding for refugee health care…” [32]		
Inequality in the healthcare system	56	10.6 %
“If the Thepens lived in British Columbia, the treatment cost would have been picked up by the province. Without a universal drug plan, Canadians face a postal-code lottery.” [33]		
Healthcare policy must balance access and budget needs	43	8.1 %
“The original lawsuit, brought by 29 families with autistic children, drew a heated rebuke from Mr. McGuinty who said he was concerned by a court ruling that requires a government to spend money it might not have.” [34]		
Health expenses are a burden on family, patient	43	8.1 %
“A breast cancer patient has had to take out a line of credit to pay for a $50,000-a-year drug because her tumour—caught through a mammogram – was a smidgen too small to qualify for the medicine.” [35]		
Debate, controversy over treatment	33	6.2 %
“Some provinces, such as British Columbia, have also refused to approve [HIFU], saying the data are not strong enough to allow the procedure to be done, even in the private sector. But it is approved and funded in some European countries such as England and Germany.” [36]		
Saves money, further health issues in the long-term	29	5.5 %
“This would decrease the future costs on the health-care system caused by multiple births and spare infertile couples the health risks associated with having twins, triplets and quadruplets, said Bouzayen.” [37]		
Making necessary drugs/treatment affordable for patients	18	3.4 %
“Hundreds of Ontario cancer patients, including many suffering from the most advanced and deadly versions of the disease, will have access to four costly new cancer drugs to be financed at public expense, the provincial government announced yesterday” [38]		
Right to access healthcare in language of choice	16	3.0 %
“An English-speaking Cornwall-area woman intends to complain to the Ontario Human Rights Commission after being refused treatment at a Cornwall francophone health centre.” [39]		

### Increasing access to healthcare in Canada

Articles were coded for whether or not there was discussion that supported increased access to healthcare. An overwhelming 513 articles (96.8 %) provided some reason or discussion about why a specific healthcare issue needs to be addressed, whether it is covering certain prescription drugs or improving the availability of certain treatments, or why access needs to be improved in general. The reasons why access needed to be improved were commonly reported to be due to various financial reasons (e.g., patient cannot afford treatment) (213 articles, 40.3 %) or that patients’ conditions will deteriorate and in some cases result in death, without greater access to healthcare (205 articles, 38.7 %). In contrast, only 178 articles (33.6 %) provided any discussion that questioned increased access. The reasons for opposing increased access also included economic factors (e.g., provincial budget limitations) (88 articles, 16.6 %) and the limited evidence of safety and/or efficacy of a certain treatment or medication (52 articles, 9.8 %). Only ten of the articles (1.9 %) did not provide any support or opposition regarding access to healthcare.

To provide an additional perspective on the portrayals of access issues, news articles were also coded for any evidence that was provided to support claims and if anyone was quoted within the articles. Evidence, which included empirical and anecdotal evidence, was referenced in articles that included support for access in the majority of articles (474 articles, 89.4.0 %), but was limited in articles that included opposition to access (107 articles, 20.2 %). Also, not surprisingly, patients were an important voice in the articles that supported increased access to healthcare, with 46.4 % of these articles containing quotations from patients. Government officials tended to be the voice represented in articles that opposed increased access (51.6 %).

### Tone of news articles

**Fig. 1:**
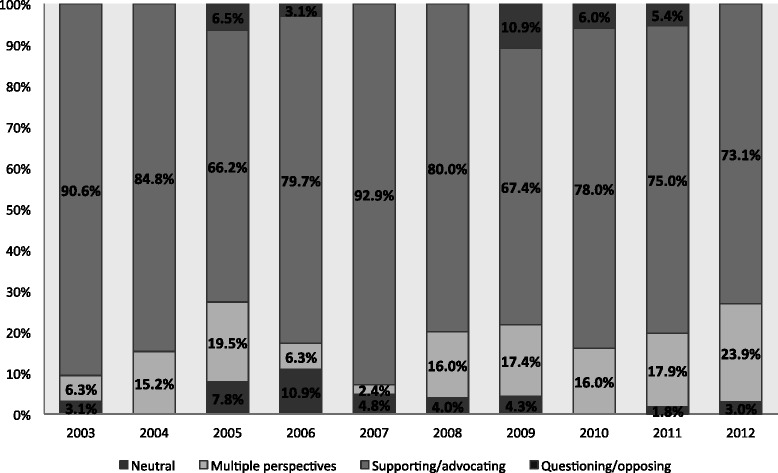
Tone of news articles each year

The most telling part of this analysis concerns the tone of the articles. Given the immense focus on patient perspectives, support for increasing access to healthcare, and the common themes of needing to maintain or improve access to healthcare and the government letting its citizens and residents down, it should not be surprising that, overall, a majority of articles were in support of increasing or improving the access to health therapies and technologies in Canada (410 articles, 77.4 %). Only 18 articles (3.4 %) were in opposition overall to the access issue highlighted in the article. It should be noted, though, that 79 articles provided multiple perspectives (79 articles, 14.9 %) or were neutral in regards to the access issue highlighted in the article (23 articles, 4.3 %). This trend did not change much throughout the decade of news coverage (Fig. 1).

## Discussion

As hypothesized, the Canadian print news media was overwhelmingly sympathetic towards patients and increasing government funding for medication, procedures, and other treatments. The majority of news coverage, regardless of issues raised, highlighted patients’ perspectives and the difficulties they face without more funding, more research, or improved access policies. Given that rare diseases and orphan drugs were mentioned most frequently in Canadian newspaper articles, these results underline the importance of access issues that concern rare diseases and orphan drugs and the potential policy impact of media coverage of these issues. However, even access issues that do not necessarily include patient cost concerns consistently favored the patients’ perspective, such as access to healthcare in language of choice and concerns over the length of the drug review process. While some debate over the economic and ethical feasibility of access did appear in the news coverage, it was largely associated with a few particular health issues that are generally portrayed (rightly or not) as a lifestyle choice, not a medical necessity (e.g., government-funded IVF in Quebec and Ontario [28]).

The results of our study support the view that Canadian news media favors a patient access ethos that supports the need to increase funding and availability of medical therapies and technologies. Given the existing literature on the role of the media in framing public policy debate, this reality may make it more difficult for other perspectives, such as a more evidence-based approach, to influence funding decisions. Policy-makers may find it difficult to resist the power of high-profile patient narratives and [6, 29], as a result, the sway of other factors, such as empirical evidence about efficacy and safety, may be inappropriately diminished. Based on previous research that has demonstrated the impact of media on policy debates and allocation policies, the almost unwavering sympathy towards patients in Canadian news coverage over the past decade raises questions about the impact of this coverage on past and future policies, including those that concern rare diseases and orphan drugs [13]. In this regard, future research should explore the relationship, if any, of media coverage and health policy decisions. Is it true that it is the squeaky wheel that gets the grease? Additionally, since it has also been shown that media both reflects and shapes public sentiment, it is likely that a media that is largely, and almost solely, sympathetic towards patients also demonstrates that there may be a strong “rule of rescue” ethos within the Canadian public, which makes it even more difficult for policy-makers to “resist the rule of rescue imperative” [8].

There are limitations to our study, but these pose opportunities for further research. First, we only looked at newspaper coverage from major print newspapers. Further research on other forms of popular media such as magazines, blogs, and social media may yield additional perspectives on issues of access in Canada. While our search terms were selected to elicit a wide range of issues, our data set highlights the media portrayals of known access issues in Canada. Second, a comparison of media coverage with governmental healthcare coverage and health policies may provide further insight into whether there are any correlations between media attention and policy-makers ability to resist the power of the rule of rescue. Finally, our selection criteria produced a data set of articles that was largely focused on conflicts between patients and government funding policies. A search that includes other access issues, such as the funding policies around rare diseases, may highlight additional themes.

## Conclusion

We understand why the patient perspective gets so much attention and sympathize with the plight of people facing real and serious health concerns that may be exacerbated by a lack of access. But our study reveals some of the challenges associated with communicating the relevant justifications associated with health policy decisions. While Tommy Douglas encouraged us to fight for the universality of healthcare in Canada, it is also important to build a system that would see Canadians receiving quality healthcare based on transparent policies that are rooted in good evidence and rational and appropriately informed debate.
